# Quantification and Analysis of Residual Stresses in Braking Pedal Produced via Laser–Powder Bed Fusion Additive Manufacturing Technology

**DOI:** 10.3390/ma16175766

**Published:** 2023-08-23

**Authors:** František Fojtík, Roman Potrok, Jiří Hajnyš, Quoc-Phu Ma, Lukáš Kudrna, Jakub Měsíček

**Affiliations:** 1 Department of Applied Mechanics, Faculty of Mechanical Engineering, VSB-Technical University of Ostrava, 70800 Ostrava, Czech Republic; 2 Department of Machining, Assembly and Engineering Metrology, Faculty of Mechanical Engineering, VSB-Technical University of Ostrava, 70800 Ostrava, Czech Republic; jiri.hajnys@vsb.cz (J.H.);; 3 Department of Machine and Industrial Design, Faculty of Mechanical Engineering, VSB-Technical University of Ostrava, 70800 Ostrava, Czech Republic

**Keywords:** powder bed fusion, SS316L, residual stress, hole drilling method

## Abstract

This study focuses on the experimental verification of residual stress (RS) in a 3D-printed braking pedal using the Powder Bed Fusion (PBF) method with SS316L material. The RS was measured at two representative locations using the hole drilling method (HDM) and the dividing method, which are semi-destructive and destructive methods of RS measurement, respectively. The finite element method (FEM) was used with Ansys Workbench 2020R2 and Simufact Additive 2021 software to determine the magnitude of RS. The results provide insights into how RS is incorporated into metal 3D-printed components and the available tools for predicting RS. This information is essential for experts to improve the accuracy and functionality of SLM parts when post-subtractive or additive manufacturing processes are used. Overall, this study contributes to the advancement of knowledge on the effects of RS on 3D-printed metal components, which can inform future research and development in this area.

## 1. Introduction

Metallic 3D printing has shown its capability in producing parts with complex geometries [1,2,3,4,5], revolutionizing the design and optimization of functional metallic components [6,7,8,9]. However, 3D-printed metal parts manufactured through Powder Bed Fusion (PBF) encounter two primary challenges: surface texture resulting from layer stacking [10,11,12], and residual stress (RS) caused by the rapid heating and cooling process [13,14].

In terms of surface texture, numerous studies have aimed to reduce the roughness of 3D-printed metal parts for both functional and aesthetic purposes, employing mechanical abrasive approaches [15,16]. Moreover, water jet peening has been explored to modify the surface texture, enabling improved cell growth for implant applications [17]. Additionally, this technology can create a desirable layer of hardened surface with RS on the component [18]. Recent studies have focused on the surface treatment of 3D-printed metal components, demonstrating that with suitable treatment methods, it is possible to enhance not only the surface texture, but also the RS profile on the component’s surface. Surface improvement is for functional and aesthetic purposes, while changing the RS profile can strengthen the part and prolong the lifetime if utilized appropriately.

As opposed to the RS introduced by mechanical work with surface treatment, a major source of RS in 3D-printed metal components is from the thermal process the manufacturing method endures. RS is a significant factor that affects the structural properties and performance of components produced through additive manufacturing [19,20]. These stress states can have a pronounced impact on the mechanical properties and behavior of printed parts, making them an important aspect that needs to be thoroughly investigated and analyzed [21]. This article focuses on the experimental verification of RS in a 3D-printed braking pedal using the Powder Bed Fusion (PBF) method with SS316L material. Under working conditions, the component is highly stressed and may undergo severe deformation under dynamic loading. Therefore, for the 3D-printed replica, it is necessary to determine the RS distribution for subsequent strength analyses and computational model tuning.

The main objective of this study is to measure the RS at two representative locations of a braking pedal using the hole drilling method (HDM) and the slicing method, which are semi-destructive and destructive methods of RS measurement, respectively. The finite element method (FEM) was employed using Ansys Workbench 2020R2 and Simufact Additive 2021 software to determine the magnitude of these stress states. The results of this study provide detailed insights into how RS is incorporated into 3D-printed metal components and the available tools for predicting these stress states. The distribution and magnitude of RS in the 3D-printed braking pedal were determined through experimental measurements and numerical analysis. The experimental measurement of RS was performed using a semi-destructive drilling method following the methodology outlined in ASTM E837-20 [22]. Additionally, a destructive cutting method was employed. The two methods were chosen because of their availability at the authors’ institution and their expertise in performing the measurements for research and industry applications.

The numerical simulation of the RS distribution on the braking pedal was performed using the Simufact Additive 2021 computational software and the Ansys Workbench 2020R2 software environment.

## 2. Materials and Methods

The braking pedal was developed based on the basis of a previous study conducted by the team. The Renishaw-AM400 3D printer (Renishaw plc., Great Britain, Wotton-under-Edge) used using PBF printing technology, and the printing parameters can be found in [23]. The manufacturing process and the strength testing procedure were extensively documented in the same paper. In this paper, the authors report a comprehensive analysis of the RS in the printed pedal through physical tests and numerical simulations, which are discussed in detail in the subsequent subsections.

### 2.1. Residual Stress Analysis in the Braking Pedal Using the Hole Drilling Method

**Figure 1:**
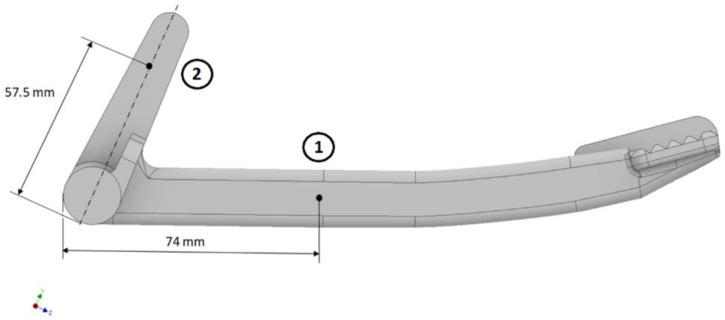
Selected locations for measuring residual stress in the braking pedal. Point (1) and (2) are for RS measurements.

HDM is a semi-destructive drilling method, according to the procedure outlined in the ASTM E837-20 standard [22], requiring the attachment of strain gauges to the surface of the measured component. Therefore, the selection of measurement locations must allow their seamless installation. For the RS analysis, the two locations in Figure 1 are chosen.

The locations of the selected points are where the strain gauges can be well positioned. Furthermore, these locations are sufficiently distant from shape changes on the pedal. They are two distinct points in the printing strategy. Point 1 is close to the supports during printing, and these supports, when removed, can influence the redistribution of RS. On the other hand, point 2 is not affected by supports. These locations are suitable for comparing computational methods with experimental results.

The RS in the braking pedal was evaluated up to a depth of 1 mm, considering both uniform and non-uniform RS. The measurement and subsequent evaluation of RS in the braking pedal were performed according to the methodology specified in the ASTM E837-20 standard. For the experimental measurement of RS using the drilling method, strain gauges CAE-06-062UL-120 [24] were utilized along with the appropriate accessories. The drilling was carried out to the desired depth using equipment compliant with the ASTM E837-20 standard.

**Figure 2:**
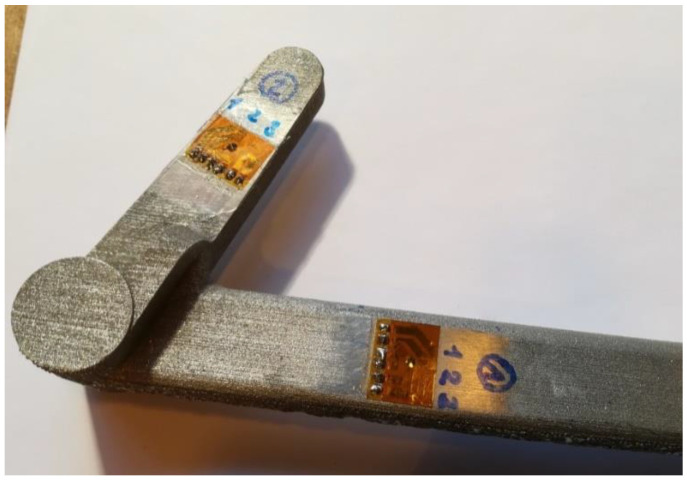
Attached strain gauges on the braking pedal at the selected measurement locations. Measurement point (1) and (2) can be observed with drilled holes from HDM.

Prior to attaching the strain gauge to the desired location, the surface roughness of the pedal was carefully modified using fine manual grinding to ensure that the values of the surface RS were not influenced by the surface roughness of the measured component [25]. After attaching the strain gauge, the drilling setup is established. The strain gauges at the selected measurement locations on the braking pedal are shown in Figure 2.

Before each measurement, it is necessary to set the null depth for each strain gauge. The drilling of the hole into the pedal is performed intermittently in specified steps [26,27,28,29]. In each step, the deformation released from each strain gauge is recorded. As part of the analysis, the distribution of uniform and non-uniform stress was assessed according to the relevant ASTM E837-20 standard.

### 2.2. Residual Stress Analysis in the Braking Pedal Using the Sectioning Method

The sectioning method belongs to the destructive RS measurement methods [30]. The measurement of RS in a specimen is based on dividing the examined body into sections or creating various arranged grooves within it. When the sample is sectioned, RS is released, resulting in the deformation of the cut sections. Depending on the type of RS, the cut sections move apart or come closer to each other. The magnitude of RS is calculated from the resulting deformations of the test sample. The FEM can be employed to determine the magnitude of RS. In this case, the computational software Ansys Workbench 2020R2 was used. This method is commonly used as a quick comparative test for quality control in material production. Furthermore, this method can be utilized for assessing RS in thin-walled tubes [30,31,32,33] and other mechanical components.

### 2.3. Simulation of Residual Stress in the Braking Pedal Using Computational Methods

The values and distribution of RS in the printed part can be determined through experimental measurements or, alternatively, by conducting a simulation of 3D printing using computational software. One of the options is to perform a 3D printing simulation within the Ansys Workbench computational program. The goal of the 3D printing simulation is to predict the deformation of the printed part and to estimate the magnitude and distribution of RS. Knowledge of deformation and RS helps prevent the destruction of the printed part during operation, thus reducing prototyping costs. The Ansys Workbench software 2020R2 incorporates the Additive Manufacturing system module, which allows the creation of a simulation workflow for additive manufacturing. The Additive Manufacturing system (AM) module [34] combines thermal analysis with structural analysis. It includes component orientation, support structure generation, and simulation of the entire printing process. This module needs to be added to the Ansys Workbench software as an extension through the Additive Wizard. Alternatively, the Ansys Additive software can be utilized for 3D printing simulation. This software is suitable for predicting RS and deformation after printing or optimizing the printing process [35].

Simufact Additive 2021 is software developed for simulating metal-based additive manufacturing processes. The program includes a database of commonly used metals, ranging from titanium and stainless steel 316L to aluminum. Users can manually input materials into the program’s database. In addition to the material database, the software also provides a database of 3D printers. It allows the simulation of four different manufacturing processes: Metal PBF, Metal binder jetting, Geometry inspection, and Machining. Simufact Additive 2021 enables the simulation of 3D printing technologies such as Selective Laser Melting, Selective Laser Sintering, and Direct Metal Laser Sintering. At the end of the 3D printing simulation, the deformation of the printed part can be evaluated. Furthermore, the software enables the prediction of the distribution and magnitude of RS introduced into the printed part during the 3D printing simulation [36].

## 3. Results

### 3.1. Resulting Values of Residual Stress in the Braking Pedal Using the Hole Drilling Method

HDM allows us to determine uniform or non-uniform stress in the body.

#### 3.1.1. Calculation of Uniform Stress

From the measured deformation values, it is possible to determine the magnitude and direction of the principal stresses σ_1_ and σ_2_, as well as the von Mises equivalent stress σ_VMS_ for uniformly distributed RS throughout the depth of the drilled hole for both measurement points identified as numbers 1 (74 mm) and 2 (57.5 mm). As aforementioned, these are the suitable locations for installing the strain gauge rosette. Location 1 is on a part of the pedal that is constrained by supports during printing. The dimensions mentioned, 74 mm and 57.5 mm, resulted from the precise position of the installed strain gauges and were transferred to the computational model for evaluation. Furthermore, it is possible to calculate the axial stress based on the orientation of the strain gauge. Table 1 presents the values of normal stresses σ_a_(1) corresponding to the normal stress in the direction of strain gauge 1 and σ_c_(3) in strain gauge 3 of the strain gauge rosette (as per the orientation indicated in Figure 2), along with the magnitude of the equivalent stress σ_VMS_ for both measurement points.

#### 3.1.2. Calculation of Non-Uniform Stress

In real bodies, non-uniform stress is commonly present. To determine non-uniform stress, a blind hole is drilled, and its depth is gradually increased in increments of 0.05 mm. Several methods can be used for calculation [37]. In order to determine the values of non-uniform stress from the released deformations, the procedure specified in ASTM E837-20 was employed. The resulting values for non-uniform RS at both measurement points are presented in Table 2.

### 3.2. Residual Stress Analysis in the Braking Pedal Using the Sectioning Method

**Figure 3:**
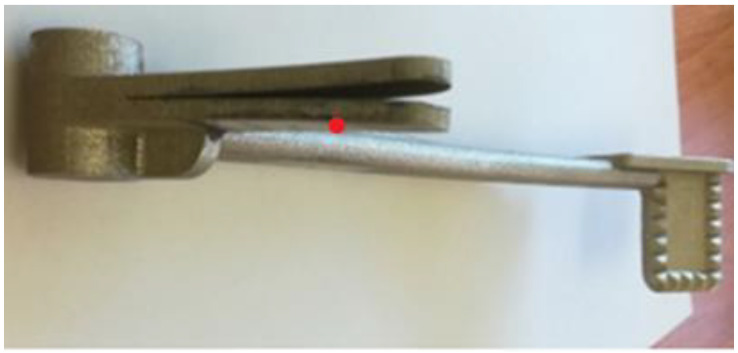
Deformation of the braking pedal after cutting it at measurement location No. 2 (red point).

The second experimental method used to measure RS in the braking pedal was the sectioning method. The length of each cut was such that it passed through the selected measurement locations. As a result of RS, the cut halves of the braking pedal experienced deformation. Figure 3 illustrates the deformation of the braking pedal caused by RS after making a cut at measurement location No. 2 (57.5 mm).

The calculation of RS σ_a_ was performed using the FEM in the Ansys Workbench computational program. The calculation of RS was carried out as a reverse problem. Boundary conditions were applied to each half of the pedal arm to achieve the same opening displacement as measured after cutting. The gap between the halves in Ansys had to match the measured gap on the actual braking pedal. Subsequently, the stress σa was evaluated at both measurement locations. The values of stress σ_a_ for both measurement locations as a function of the depth from the outer surface are provided in Table 3.

### 3.3. Analysis of Residual Stress in the Braking Pedal Using the Ansys Workbench 2020R2 Computational Program

**Figure 4:**
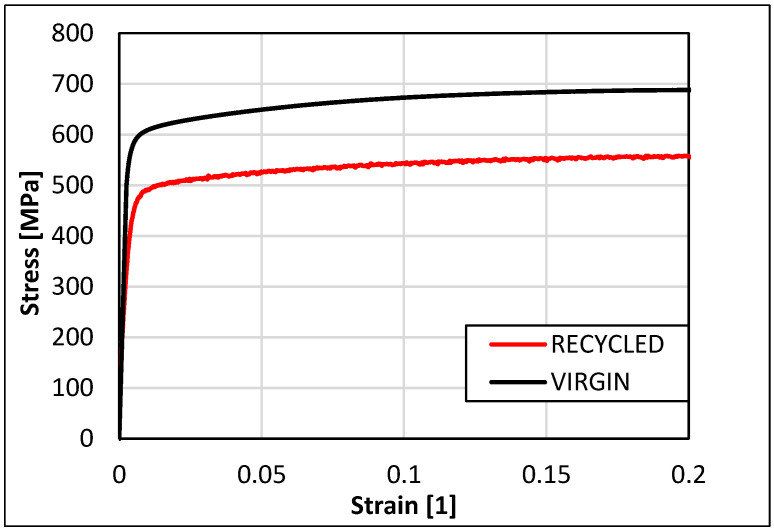
Stress–strain curves of the original and recycled powder material of stainless steel 316L during the tensile test [23].

The Ansys Workbench 2020R2 computational program features a database of commonly used materials for 3D printing. For the production of the braking pedal, recycled powder material of stainless steel 316L was chosen because it is the most common 3D-printed material with a well-established knowledge base regarding the printing and treatment, as reviewed in the Introduction. Therefore, it was necessary to determine the corresponding material parameters of the recycled material before simulating the 3D printing process and adjust them in the Ansys Workbench 2020R2 program. A tensile test was performed on the original and recycled powder material of stainless steel 316L, revealing differences in the values of yield strength and ultimate tensile strength. Tensile tests were performed at room temperature and are shown in Figure 4 [23].

**Figure 5:**
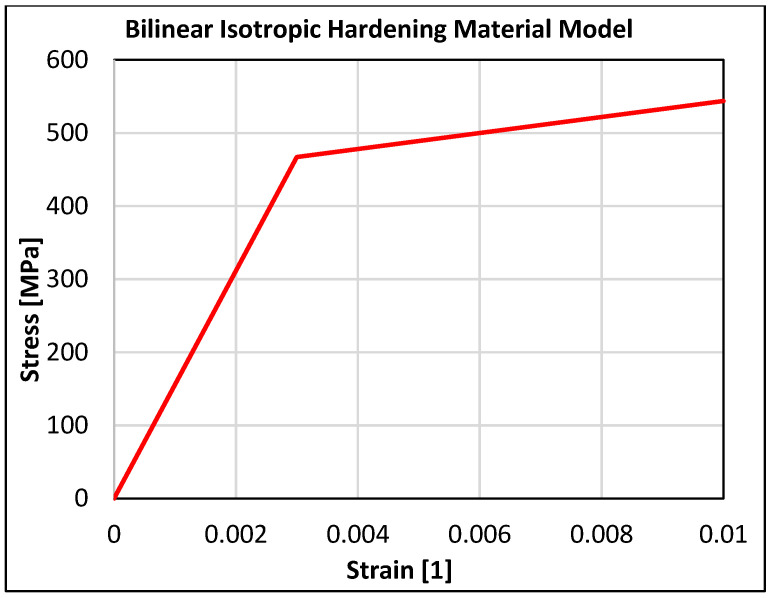
Bilinear isotropic hardening model of the recycled powder material of stainless steel 316L.

The data were taken from the average of five tests. Tensile samples were vertically distributed in the center of the building platform, where the braking pedal was to be printed following the same setup. The virgin powder was first used for printing, and then it was filtered to eliminate the melted, but unsintered particles. Then, it was mixed with virgin powder in a 1:1 ratio. This is one cycle of recycling. The braking pedal in this study was printed with powder recycled five times. The yield modulus of elasticity (E) was determined from the stress–strain curve of the tensile test for the recycled powder material of stainless steel 316L. Its value was used to construct a bilinear isotropic hardening model. The assembled bilinear hardening model for the recycled powder material of stainless steel 316L at a temperature of 22 °C is shown in Figure 5.

The analysis of RS distribution in the printed braking pedal was performed using the inherent strain method. In contrast to Simufact Additive 2021 software, the Ansys Workbench 2020R2 program does not require specific values of inherent strain. Instead, a strain scaling factor (SSF) needs to be set [38], which can be understood from Equation (1), where *σ_Y_* represents the yield strength (MPa) and *E* is the tensile modulus of elasticity (MPa). Additionally, the layer thickness was set to 50 µm.

(1) $$\varepsilon =SSF∙\frac{\sigma_{Y}}{E},$$

**Figure 6:**
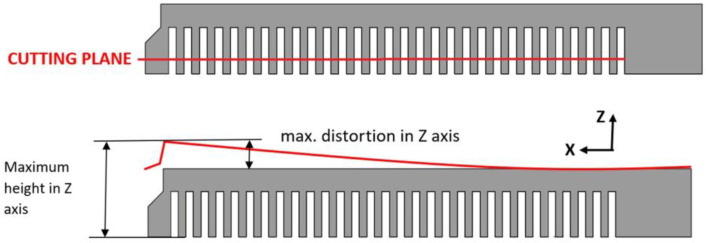
Measurement of the deformation of the test specimen [31].

Prior to simulating the printing process in the Ansys Workbench 2020R2 software, a calibration procedure is necessary, as detailed in the software manual [38]. The goal of calibration is to ensure that the simulated printing of the component in the software corresponds to the actual printed part. Calibration is performed by physically printing a cantilever specimen. Simultaneously, the same cantilever is simulated using Ansys Workbench 2020R2 software. After printing, the test specimen is cut at a height of 3 mm from the build plate, and the height of the cut end relative to the build plate along the Z-axis is measured, representing the deformation caused by RS (Figure 6). Subsequently, the measured value is compared with the deformation value of the cantilever obtained from the simulation of the test specimen printing.

**Figure 7:**
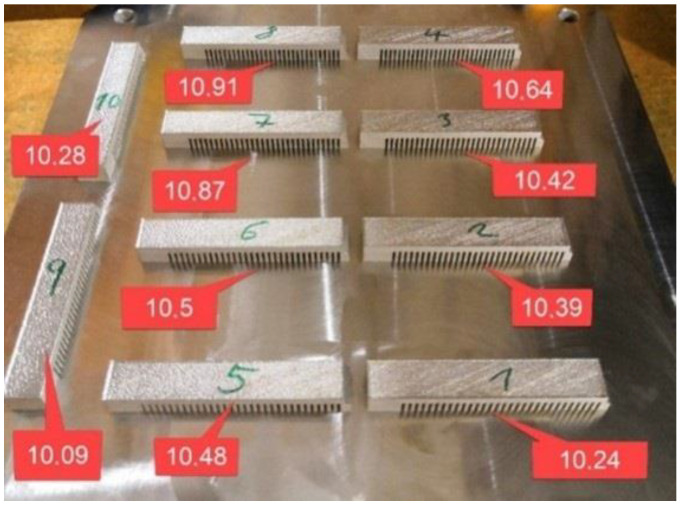
Placement of the test specimens on the build plate and their deformations.

Ten test cantilevers were printed for calibration, and they were placed at various locations on the build plate. Subsequently, the distance between the end of each cantilever and the build plate was measured for all test specimens (Figure 7). The distance of the deformed ends of the cantilevers in the Z-axis ranged from 10.09 mm to 10.91 mm.

In Ansys Workbench 2020R2, a computational model of the cantilever was created, with the same dimensions as the physically printed cantilever. Subsequently, a 3D printing simulation was conducted using inherent strain. Calibration was achieved with multiple cantilevers distributed at different positions on the building platform to record the average inherent strain. In addition, the tensile specimens and the braking pedal were printed and loaded in the same direction, ensuring that the strength analysis was valid.

**Figure 8:**
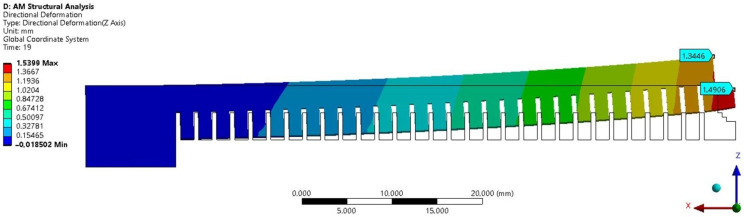
Deformation of the cantilever end after the 3D printing simulation in Ansys Workbench. Point a and point b distort in Oz direction 1.3446 mm and 1.4906 mm, respectively.

After cutting the cantilever from the build plate, the deformation of the printed cantilever was compared to the simulated deformation (Figure 8). The goal was to determine the optimal value of the strain scaling factor (SSF) parameter. If the deformations do not match, new values of the SSF parameter need to be set [38]. Due to the wide range of actual measured deformations, finding the exact value of the SSF parameter was computationally demanding. This is because for the calibration of the cantilevers to obtain inherent strains, we had to prescribe ten values of z deformation in correspondence with ten cantilevers at the exact position on the build plate, as in reality. Then, the software will have to find one set of inherent strains to satisfy the deformation of ten such values.

For the 3D printing simulation of the braking pedal, the value of SSF was set for anisotropic material. The SSF parameters used for each direction are listed in Table 4.

**Figure 9:**
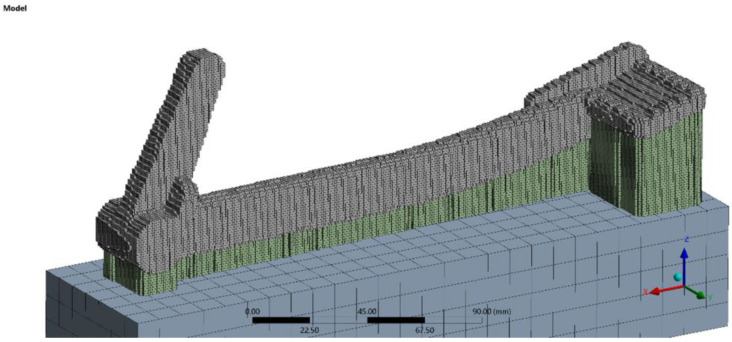
Computational model of the braking pedal in Ansys Workbench.

To simulate the 3D printing of the braking pedal, it is necessary to create a corresponding computational model represented by voxels. The resulting computational model has the parameters listed in Table 5, and a visual representation of the computational model can be seen in Figure 9.

In Ansys, a local coordinate system was set at measurement point 2 (57.5 mm), aligned with the axes of the strain gauges of the strain rosette. The 3D printing of the Indian Scout motorcycle braking pedal in Ansys Workbench was performed under the conditions specified in Table 6.

**Figure 10:**
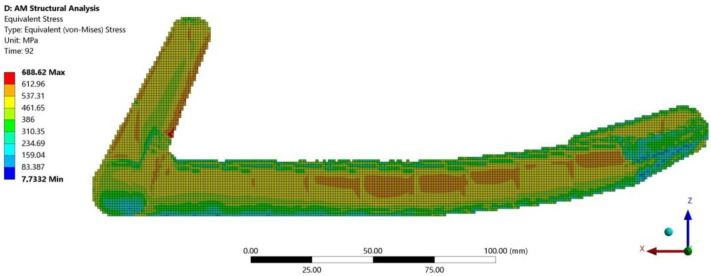
Distribution of equivalent stress in the braking pedal.

The distribution of equivalent stress after printing the braking pedal and its subsequent removal from the build plate and support removal is shown in Figure 10.

The magnitudes of normal stresses σ_a_(1), σ_c_(3), and von Mises stress distribution as a function of depth from the outer surface at measurement point 1 and measurement point 2 obtained from the numerical analysis in Ansys Workbench are presented in Table 7.

### 3.4. Residual Stress Analysis in the Braking Pedal Using the Simufact Additive 2021 Computational Program

The computational analysis of RS was also conducted in the Simufact Additive 2021 program [36]. In our case, the mechanical method was used, which requires determining the value of the inherent strain. Prior to printing, calibration is also necessary, and its detailed procedure is provided in the Simufact Additive 2021 software. The goal of calibration is to ensure that the simulation of the printing process in the computational program corresponds to the printing of the actual part. Calibration is performed by printing a test sample of a cantilever that was identical to the one used for calibration in the Ansys Workbench program. The aim of calibration in Simufact Additive 2021 is to determine the value of inherent strain for all directions, and the resulting values are presented in Table 8.

To simulate the printing process, it was necessary to create a computational model from the geometric model of the braking pedal. In this case, the computational model consists of voxels. To print the desired part, it is necessary to model the supports, which can be created separately and imported into the computational program before printing or the supports can be automatically generated by the program. For the simulation of the braking pedal printing, supports were automatically generated by the Simufact Additive 2021 software. The parameters of the resulting computational model are provided in Table 9.

Before simulating the 3D printing process in Simufact Additive 2021, it is necessary to set the printing parameters. The same material parameters used in the simulation of 3D printing in Ansys Workbench (Table 6) were applied for the printing. Additionally, based on the selected 3D printing simulation method, it is important to configure the printing parameters and printer settings, which are provided in Table 10.

**Figure 11:**
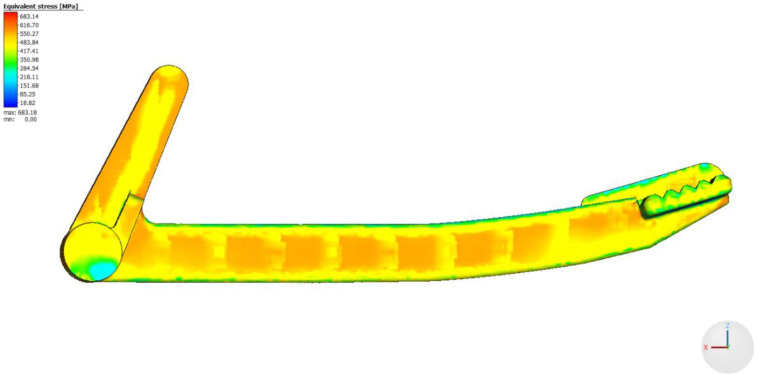
Distribution of equivalent stress in the braking pedal in Simufact Additive 2021.

The distribution of RS in the printed part can be evaluated in Simufact Additive 2021 on the build plate, including the supports. Furthermore, it is possible to analyze the distribution and magnitude of RS after removing the printed part from the build plate, but before removing the supports. The last option is to evaluate RS after removing both the printed part and the supports from the build plate. Since the experimental measurement of RS was conducted on the braking pedal without supports, the last option was used for the analysis of RS in the braking pedal. The distribution of equivalent RS according to the von Mises theory in the braking pedal is shown in Figure 11.

**Figure 12:**
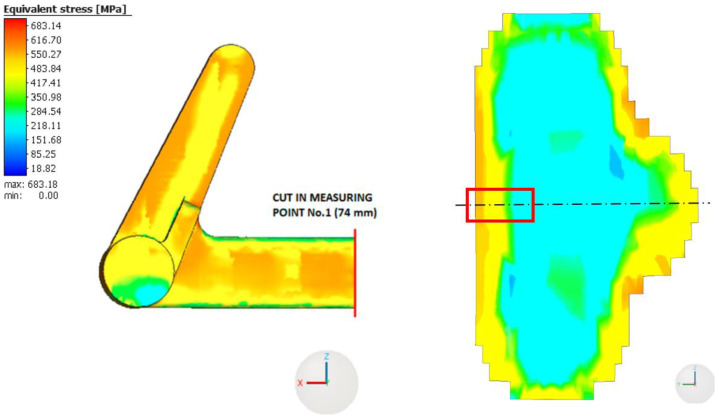
Distribution of the equivalent residual stress at measurement point No. 1 (74 mm).

**Figure 13:**
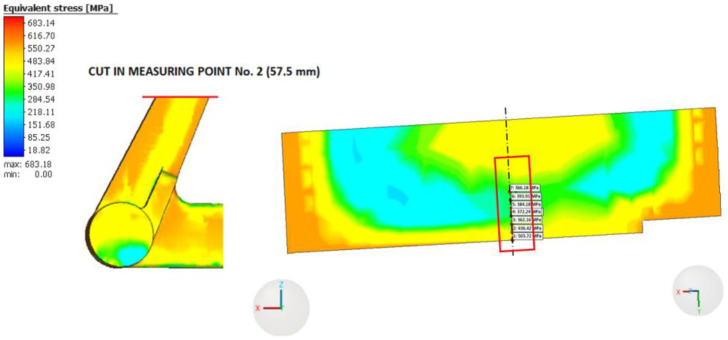
Distribution of the equivalent residual stress at measurement point No. 2 (57.5 mm).

The highest values of equivalent RS are located on the surface of the braking pedal and in its immediate vicinity. As we move from the surface towards the center of the braking pedal, the magnitude of RS gradually decreases, as can generally be observed in Figure 12 and Figure 13. The distribution and magnitude of the equivalent stress at measurement point No. 1 (74 mm) are shown in Figure 12.

The distribution of equivalent RS at measurement point No. 2 (57.5 mm) at a distance of 57.5 mm is shown in Figure 13.

At this measurement point as well, it can be observed that the highest values of equivalent RS are attained on the surface of the braking pedal and in its immediate vicinity. The equivalent RS in the braking pedal initially decreases as we move from the surface towards the center, but at a distance of approximately 2 mm, the magnitude of the equivalent RS starts to increase again. It is likely related to the layer effect (across the layers in Figure 12 versus on one layer in Figure 13) and the distribution of the temperature field during printing and subsequent cooling.

Table 11 provides the numerical values of normal RS σ_a_(1) and σ_c_(3), as well as the values of the equivalent stress σ_VMS_ at both measurement points. The graphical interpretation of the stress distribution at the measurement points for selected depths is presented. A comparison with the experimental results will be conducted in the following subsection.

### 3.5. Comparison of the Achieved Residual Stress Results in the Braking Pedal for All Methods and Both Measurement Points

The following graphs compare the RS values obtained from experimental measurements (HDM, sectioning method) with the values of RS obtained from the simulation of the 3D printing of the braking pedal in the software programs Simufact Additive 2021 and Ansys Workbench 2020R2 at both measurement points. Since the destructive sectioning method was also used to determine the values of RS, the comparison will be made between the values of equivalent RS σ_VMS_ and residual normal stresses σ_a_(1) and σ_c_(3).

**Figure 14:**
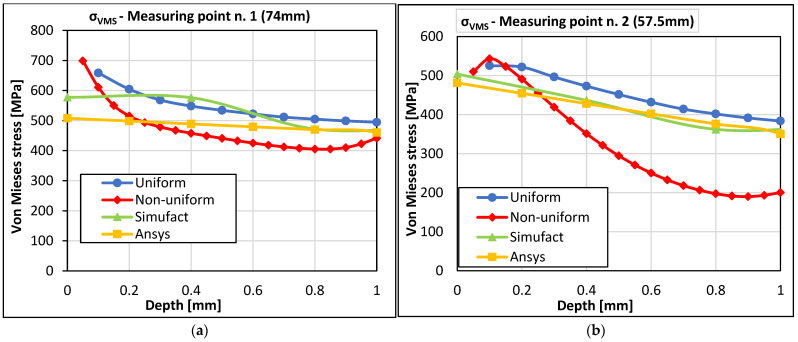
Mutual comparison of the results obtained from von Mises stress (σ_VMS_) (**a**) at measurement point 1 and (**b**) at measurement point 2.

**Figure 15:**
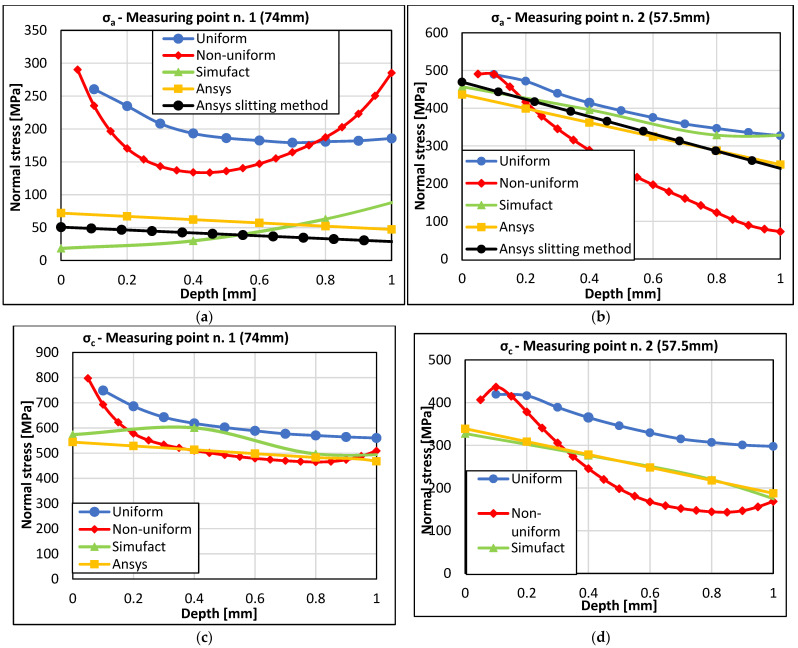
Mutual comparison of the results obtained from normal stresses (**a**,**c**) at measurement point 1 and (**b**,**d**) at measurement point 2.

In Figure 14, a comparison of the equivalent RS according to von Mises theory (σ_VMS_) is shown for both measurement points. Upon observing both graphs, we can see nearly linear trends in the values of uniform residual equivalent stress obtained from the simulation of 3D printing of the braking pedal in Ansys Workbench and Simufact Additive 2021. This fact is most pronounced in the graph for measurement point 2 (57.5 mm). As for measurement point 1, the was a remarkable step change between 0.4 and 0.8. This is likely due to the difference in the stress distribution observed experimentally and in the calculations in directions a and c (refer to Figure 15a,c). The stress measured in direction c is approximately half of that in direction a at this measurement point. During the setup of computational models, attempts were made to tune the parameters in the direction of larger residual stresses, specifically in the direction c, considering their magnitude.

The linear behavior of the RS values in the numerical solution is influenced by the size of the voxels used in the computational model of the braking pedal. Considering that the uniform stress profile obtained from the HDM also exhibits a similar linear trend, the values of equivalent stress at both measurement points will be specifically compared to the uniform stress values. It should also be noted that the drilling method used herein allows for the evaluation of RS up to a depth of 1 mm. Based on the experiences with 3D printing technology and the computational analyses presented in Figure 12 and Figure 13, it is evident that the highest RS occurs precisely at the surface and in a small depth beneath it. The authors consider a depth of 1 mm to be adequate for this purpose.

Larger differences in the values of the equivalent stress σ_VMS_ were observed on the surface of the braking pedal and in the subsurface layer, which applies to both measurement points. For measurement point 1 (74 mm), it is evident that with increasing depth, the difference between the values of uniform and non-uniform equivalent stress gradually decreases compared to the values obtained from the numerical solution. In Figure 14a, it can be seen that at a depth of 1 mm from the surface of the braking pedal, the difference between the maximum and minimum values of the equivalent stress is 52.74 MPa. The situation is different for measurement point 2, Figure 14b. The reduction in the difference between the RS values only occurs in the case of uniform equivalent stress and the values from the numerical solution. The gradient of non-uniform equivalent stress is higher up to a depth of 0.8 mm from the surface of the braking pedal. From a distance of 0.8 mm to a depth of 1 mm, the value of non-uniform equivalent RS remains nearly constant. On the other hand, it can be observed that the gradient of uniform equivalent stress and the gradients of the equivalent stress from the Simufact Additive and Ansys Workbench programs are very similar (the slopes of the lines are very close). In the case of these stresses, there is a gradual reduction in the mutual differences, and the smallest difference of 33.53 MPa was achieved at a depth of 1 mm.

Figure 15a,b show the profiles of normal stresses in the direction of strain gauge 1 at measurement point 1 (74 mm) and measurement point 2 (57.5 mm), respectively. The results obtained from the HDM for both the uniformly and non-uniformly distributed RS up to a depth of 1 mm from the outer surface are presented. Additionally, the results of normal stress obtained in this direction and the measurement point using the sectioning method are shown. Furthermore, the results of the normal RS obtained by simulation in Ansys Workbench and Simufact Additive 2021 are included. For measurement point 1 in direction 1, the stress results obtained from the sectioning method correlate well with the computed stress profile in Ansys Workbench. The results of the normal stress obtained in Simufact Additive 2021 exhibit a non-standard profile, which does not correlate with the resulting reduced stress indicated in Figure 14a. Higher RS values were obtained from the drilling method. Compared to the reduced stress values according to von Mises theory, which are comparable to the calculations, this indicates a different stress redistribution in the measured region. From a quantification perspective, the sectioning method is in better agreement with computational methods in this region. However, it should be noted that the sectioning method provides average stress values derived from the deformation of half the thickness of the crankshaft at the respective location, whereas the HDM obtains values corresponding to the specific measured depths for both methods of RS evaluation.

For measurement point 2 in direction 1, both experimental methods show relatively good agreement compared to the results obtained from both computational methods. Figure 15c,d present the results of normal RS in the direction of strain gauge 3. The results obtained from the HDM and the results obtained from numerical simulations in both software programs are shown. The sectioning method was not used in this direction. For measurement point 1, there is a very good agreement between the experimentally determined stress and the numerical simulations from a depth of 0.2 mm onward. The normal stresses obtained from HDM on the surface up to a depth of 0.2 mm are higher, which corresponds to the trend observed in 3D printing on the surface with the SLM method. For measurement point 2 in direction 3, the values of residual normal stresses obtained from the HDM are greater than the stresses obtained from numerical simulation. This difference is likely due to stress redistribution, taking into account the results of reduced stresses according to von Mises theory, as shown in Figure 14b. The results of RS obtained through measurements and computational modeling in the form of VMS stress, as shown in Figure 14, exhibit relatively good agreement. It is essential to consider that the computational models were set up based on bridge calibration. When this calibration setup is applied to a real component, it shows a reasonably good match, despite the shape differences between the calibration bridges and the actual component, the pedal.

## 4. Conclusions

From the results, it can be concluded that at the measurement point farther from the build plate or supports during printing, there is a better correlation between the calculated and experimentally determined RS profiles. This could be because of different heat dissipating efficiency at the measured points. It should be noted that the material model and the printing parameters are based on the deformation methodology of cantilevers, which are significantly smaller in size and volume compared to the printed pedal. The calibration results shown in Figure 7 indicate that the deformation of individual cantilevers varies depending on their position on the measurement substrate, indicating the influence of printing parameters, powder flow in the printing chamber, and other factors. However, in the settings of the individual programs, only the average value of this parameter is taken into account, which affects the results of both numerical simulations.

The information obtained in this study is essential for experts striving to improve the accuracy and functionality of components produced through PBF when post-subtractive or additive manufacturing processes are employed. Overall, this study contributes to advancing knowledge regarding the effects of RS on 3D-printed metal components, which can inform future research and development in this field. Given the increasing interest in additive manufacturing and the need to enhance the properties of printed components, it is crucial to gain a comprehensive understanding of the influence of RS on these complex structural arrangements. We hope that the findings of this study will contribute to improving evaluation techniques and optimizing 3D-printed metal components, thereby fostering further development in this promising field.

## Figures and Tables

**Table 1:** Calculated values of uniform stress at both measurement points.

	Uniform Stress					
Drilling Depth (mm)	Measurement Point No. 1 (74 mm)			Measurement Point No. 2 (57.5 mm)		
	σ_a_(1) (MPa)	σ_C_(3) (MPa)	σ_VMS_ (MPa)	σ_a_(1) (MPa)	σ_C_(3) (MPa)	σ_VMS_ (MPa)
0.1	261	749	659	490	420	525
0.2	235	686	604	472	416	522
0.3	209	643	569	440	389	496
0.4	194	618	548	414	365	473
0.5	187	602	534	394	346	451
0.6	183	589	522	375	329	432
0.7	180	577	512	358	315	414
0.8	181	570	505	346	307	402
0.9	182	564	499	336	301	391
1.0	186	560	494	327	298	384

**Table 2:** Calculated values of non-uniform stress at both measurement points.

Non-Uniform Stress						
Drilling Depth (mm)	Measurement Point No. 1 (74 mm)			Measurement Point No. 2 (57.5 mm)		
	σ_a_(1) (MPa)	σ_C_(3) (MPa)	σ_VMS_ (MPa)	σ_a_(1) (MPa)	σ_C_(3) (MPa)	σ_VMS_ (MPa)
0.05	290	797	699	491	407	510
0.10	235	693	611	490	436	543
0.15	197	622	550	457	415	523
0.20	170	578	515	416	378	491
0.25	154	551	494	379	341	455
0.30	143	533	479	345	306	419
0.35	137	521	468	316	274	384
0.40	134	510	458	288	246	351
0.45	134	501	449	263	220	321
0.50	136	493	441	239	199	294
0.55	140	485	433	217	181	271
0.60	147	479	425	197	168	250
0.65	155	474	418	179	159	233
0.70	165	470	413	161	152	218
0.75	175	467	408	142	148	206
0.80	187	465	405	123	144	197
0.85	203	467	405	105	143	192
0.90	223	474	410	90	147	190
0.95	250	488	423	79	156	193
1.00	285	509	442	73	169	200

**Table 3:** Values of stress σ_a_(1) at measurement location No. 1 and measurement location No. 2 obtained using the sectioning method in the Ansys Workbench program environment.

Depth (mm)	Measurement Point No. 1 (74 mm)	Measurement Point No. 2 (57.5 mm)
	σ_a_(1) (MPa)	σ_a_(1) (MPa)
0	51	469
0.1	49	447
0.2	46	424
0.3	44	401
0.4	42	378
0.5	40	355
0.6	38	332
0.7	35	309
0.8	33	286
0.9	31	263
1.0	29	240

**Table 4:** Values of the SSF parameter for the 3D printing simulation of the braking pedal.

SSF_X_	SSF_Y_	SSF_Z_
0.98	0.98	0.997

**Table 5:** Parameters of the computational model of the braking pedal.

Number of Voxels	Number of Nodes
152,057	178,462

**Table 6:** Material parameters for 3D printing of the braking pedal [23].

Parameter	Value
Material	SS316L
Yield strength	467 MPa
Tensile strength	614 MPa
Young’s modulus	204 GPa
Shear modulus	10.96 GPa
Poisson’s ratio	0.29
Layer thickness	50 µm
SSF_X_	0.98
SSF_Y_	0.98
SSF_Z_	0.997

**Table 7:** Values of normal stresses σ_a_(1) and σ_c_(3), and the von Mises stress σ_VMS_ at both measurement points from Ansys Workbench.

Depth (mm)	Measurement Point No. 1 (74 mm)			Measurement Point No. 2 (57.5 mm)		
	σ_a_(1) (MPa)	σ_C_(3) (MPa)	σ_VMS_ (MPa)	σ_a_(1) (MPa)	σ_C_(3) (MPa)	σ_VMS_ (MPa)
0.0	72	544	508	436	339	481
0.2	67	529	498	399	309	454
0.4	62	513	489	362	279	428
0.6	57	498	479	325	248	402
0.8	52	483	470	288	218	376
1.0	47	467	460	250	188	350

**Table 8:** Inherent strain used for the simulation of the braking pedal 3D printing [23].

ε_xx_ [−]	ε_yy_ [−]	ε_zz_ [−]
−0.00286296	−0.00277407	−0.03

**Table 9:** Parameters of the computational model for the braking pedal in Simufact Additive 2021.

Number of Voxels	Number of Nodes
543,098	610,580

**Table 10:** Parameters configured for the 3D printing simulation of the braking pedal in Simufact Additive 2021.

Parameter	Value
Laser power	200 W
Scanning speed	650 mm/s
Layer thickness	50 µm
Hatching distance	0.11 mm
Increment rotating angle	67°
Temperature	Room temperature
ɛ_xx_	−0.00286296
ɛ_yy_	−0.00277407
ɛ_zz_	−0.03

**Table 11:** Values of the normal stresses σ_a_(1) and σ_c_(3), as well as the equivalent stress σ_VMS_, at both measurement points obtained from Simufact Additive 2021.

Depth (mm)	Measurement Point No. 1 (74 mm)			Measurement Point No. 2 (57.5 mm)		
	σ_a_(1) (MPa)	σ_C_(3) (MPa)	σ_VMS_ (MPa)	σ_a_(1) (MPa)	σ_C_(3) (MPa)	σ_VMS_ (MPa)
0	−18	574	578	457	-	504
0.4	30	600	576	396	-	436
0.8	63	497	472	329	-	362
1.2	115	505	478	338	-	372

## Data Availability

Data available on request.
